# The Effect of Negative Pressure in the Abdominal Cavity With Suprasorb CNP on Abdominal Organs—An Experimental Study

**DOI:** 10.3389/fsurg.2020.584926

**Published:** 2021-02-10

**Authors:** Thomas Auer, Iris Wiederstein-Grasser, Siegfried Sauseng, Pavle Delcev, Karl H. Preisegger

**Affiliations:** ^1^General, Visceral and Transplant Surgery, Department of Surgery, Medical University of Graz, Graz, Austria; ^2^Biomedical Research Institute, Medical University of Graz, Graz, Austria; ^3^Institute of Morphological Analytics and Human Genetics Graz, Graz, Austria

**Keywords:** negative pressure, open abdominal therapy, Suprasorb CNP^R^, porcine model, fistula

## Abstract

Since the introduction of negative pressure therapy of the abdomen, care has been taken to protect the intestine from the effects of negative pressure in order to avoid impairments of abdominal organs. As an alternative to the widespread AB-Thera^R^ system (KCI, San Antonio, Texas, USA), the different concept of Suprasorb CNP^R^ (Lohmann & Rauscher, Austria-Germany) was introduced by the producer with the premise of achieving a better therapeutic effect. Due to numerous pores of the film, the effects of the negative pressure are brought to the surface of the intestinal organs and these effects were tested on seven experimental animals. Particular attention was paid to the small intestine, colon, liver, and pancreas. Over 8 h continuously, three animals were tested with −80 mmHg, 4 with −60 mmHg. The results showed no macroscopic pathological changes. The histological results showed borderline changes in the small intestine and colon with −80 mmHg application, minimal or none with −60 mmHg. The liver and pancreas were found free of pathological changes. For use on human organs, the intra-abdominal application of −60 mmHg for the Suprasorb CNP system is proposed as the standard.

## Introduction

The use of negative pressure (NP) dressings for open abdominal therapy has undoubtedly advanced the treatment of secondary peritonitis and abdominal compartment syndrome (1–4). Concerns were expressed since the introduction of NP-driven intra-abdominal dressings; these treatments could be the inherent source of intestinal impairments (5–8).

**Figure 1:**
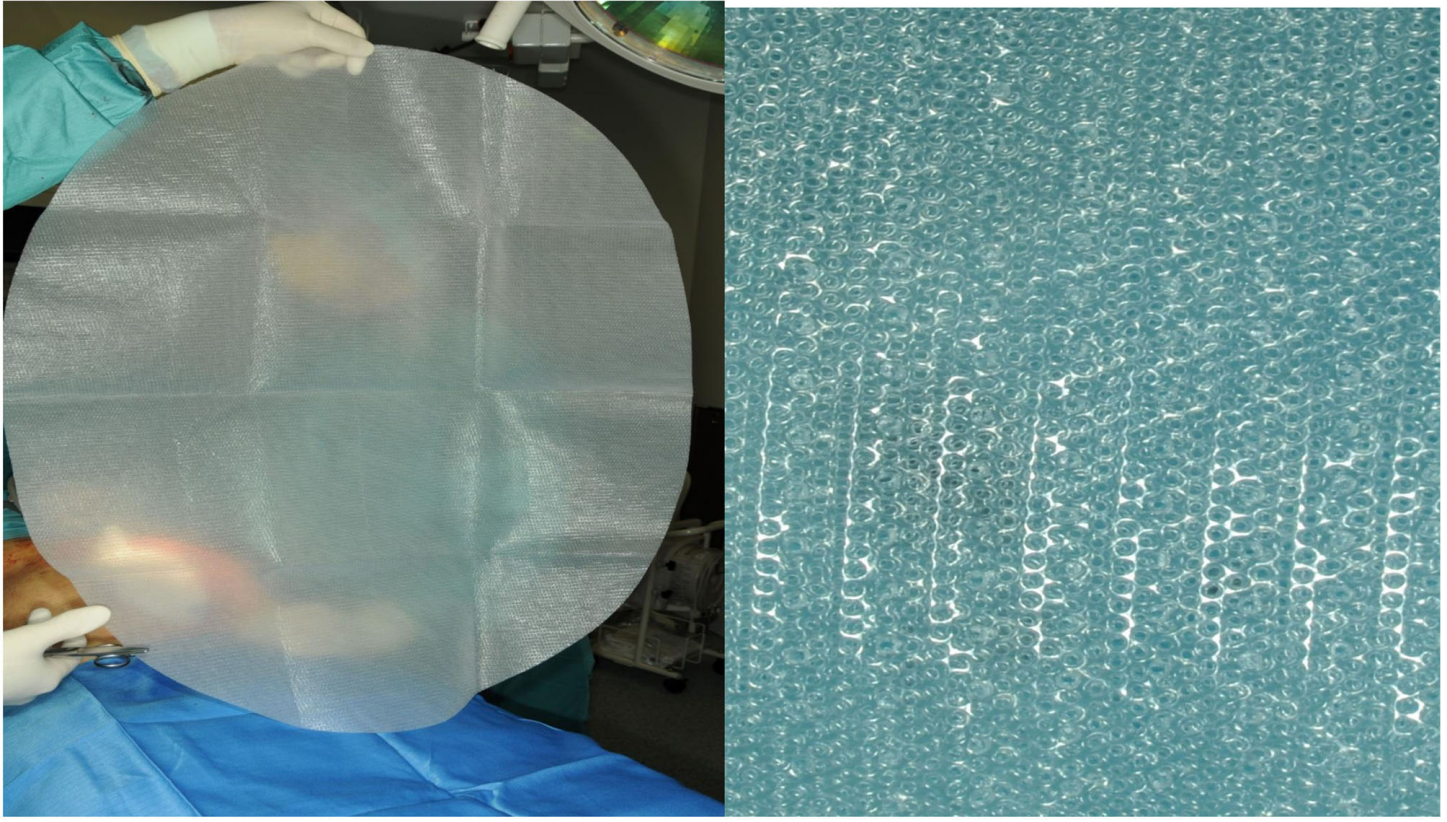
Suprasorb CNP film.

AB-Thera-V.A.C.^R^ (KCI, San Antonio, USA) is a widely used commercial system. The system uses a double-layer foil with polyurethane foam welded between. Both foils are perforated with slits to transport the fluids out of the abdomen and protect the intestine simultaneously with the nonadhesive foil on the surface. A further protective effect was thought with the fact that very low NP (−2 to −10 mmHG) of the applied −50 to −150 mmHg affects the intestine surface (9, 10). An alternative abdominal NP system was presented with the Suprasorb-CNP^R^ system (Lohmann & Rauscher, Austria-Germany). In contrast, this system uses a multiple-perforated double-layer foil (Figure 1) in direct contact with the intestinal organs with the intent to clean out the contaminated intestinal surface from both fluids and inflammatory material, as anticipated by the producer.

This large animal study was designed to elucidate the effect of full NP application on different intestinal organ structures using the Suprasorb-CNP^R^ system.

## Materials and Methods

**Figure 2:**
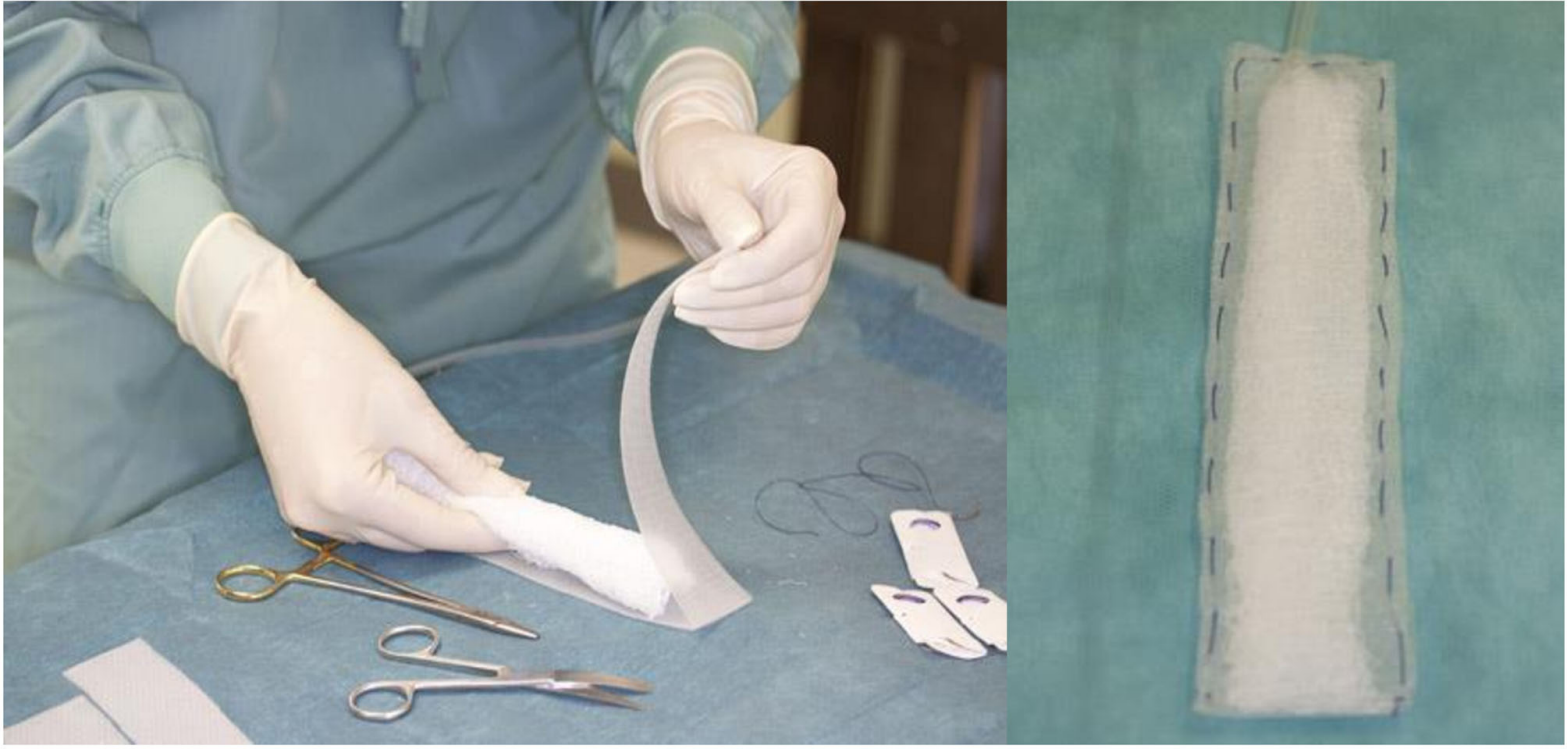
Production of the suction pads.

By Approval of the Austrian Ministry, according to the animal testing law (BGBI.Nr.501/1988 i.d.g.F.), seven domestic pigs (30–35 kg) were operated under general anesthesia. The general anesthesia was performed by an experienced specialist in animal anesthesia and the help of two animal keepers, for all animals for the entire duration. The abdominal cavity was opened with median laparotomy. Surgical procedures such as cholecystectomy, a small bowel stapler side–side anastomosis, a longitudinal colon diathermy incision, closed with a single-stitch suture line, and exploration of the pancreas surface were done. These four sites were then covered each with a suction pad. These pads were handcrafted (Figure 2), and a Jackson drain^R^ in the center was wrapped with Kerlix^R^ gauze and covered with the Suprasorb CNP^R^ film, closed around with a running suture. After suture fixation of the intestine pads and positioning of the liver and pancreas pad, the whole intestine convolute was covered with the foil. Each Jackson drain^R^ was led separately out of the abdomen, connected with the suction unit. A continuous NP was applied for 8 h, four experiments with −60 mmHg, three with −80 mmHg. This NP, well below the values of other systems, was chosen on the assumption that the full amount of NP will affect the intestine surface. After 8 h of suction, the abdomen was reopened, the surface areas were inspected, and the treated organ parts were removed for histological investigation. The histological specimens were examined both on the foil-bearing sections and on the adjacent foil-free sections. These were seen as a control group without foil therapy, with NP application only.

### Primary Endpoint

Damage to the intestine: histological findings, microcirculatory impairments, necrosis signs, damage of liver and pancreas tissue, triggering of pancreatitis. Histological findings for foil-bearing and foil-free sections.

### Secondary Endpoints

Degree of attachment of the foil, macroscopic findings: traces at all the surfaces, fistula formation of the areas of anastomosis, foil-covered and free areas, bowel surface, gall fistula formation. Suction delivery rates.

## Results

All test animals could be kept in a stable circulatory state during the entire anesthesia period; hyperthermia episodes were not observed. After 8 h of treatment and reopening of the abdominal cavity, the covering film was removed.

### Secondary Endpoints (Macroscopic Appearance)

**Figure 3:**
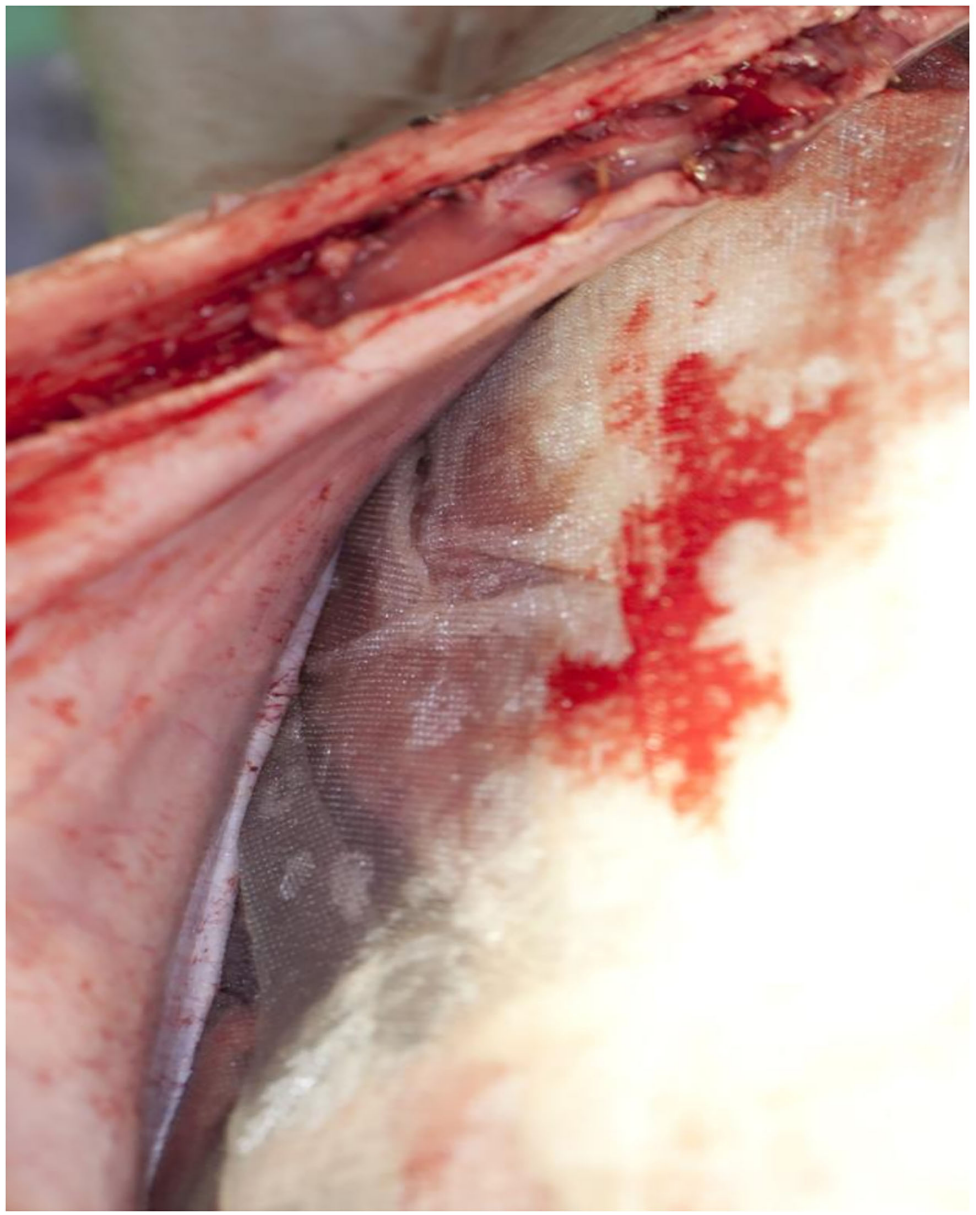
The Suprasorb-CNP film easily detached from the abdominal wall.

**Figure 4:**
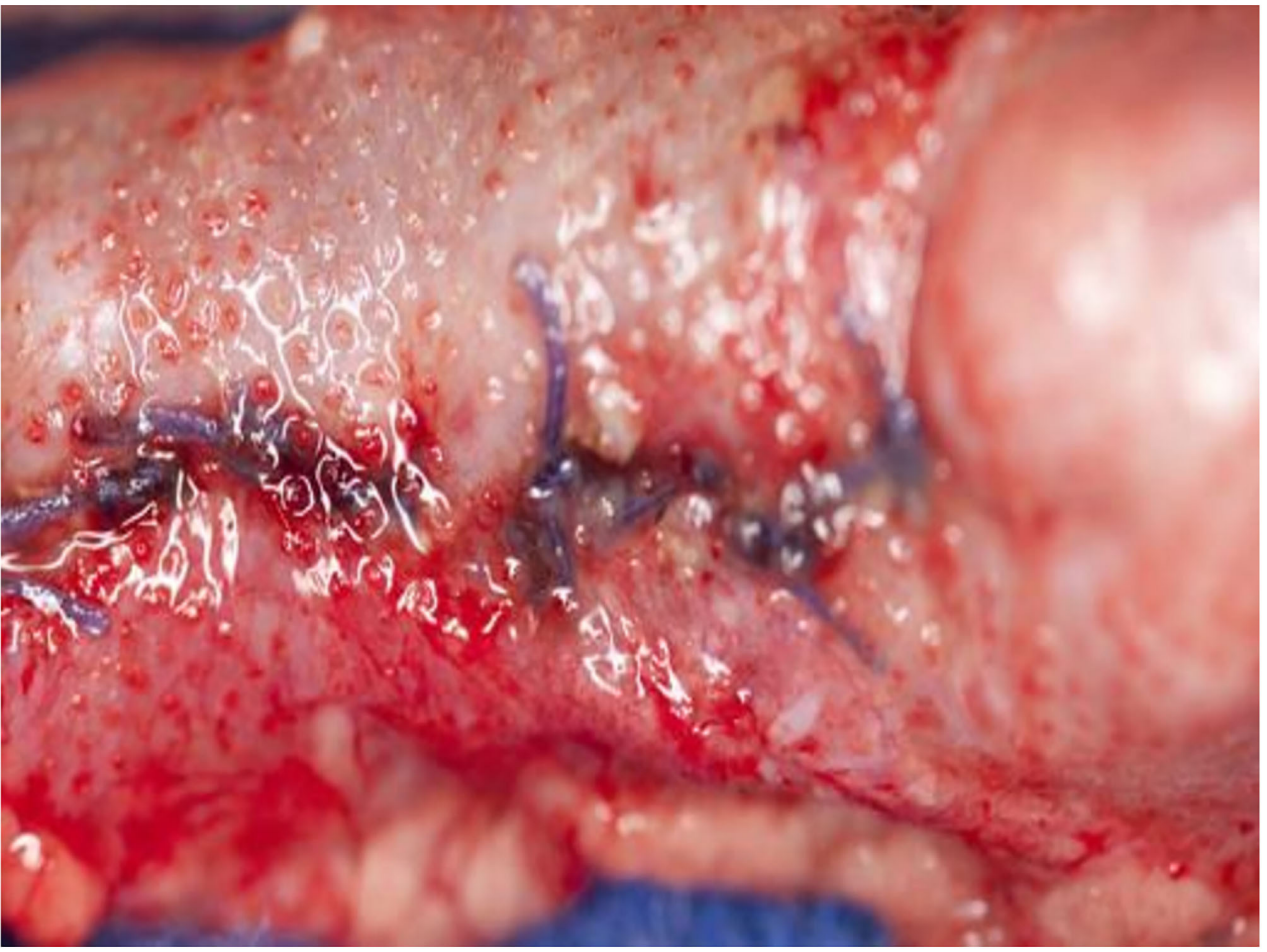
Colon defect closed with single sutures after 8 hours of NP application.

The film was easy to remove without any visible impairment on all animals whether treated on −80 or −60 mmHg (Figure 3). There was no fluid accumulation found in any animal, no visible sign of damage or traces of discolored fluids, and no signs of fistula formation on the whole bowel surfaces. The mean amount of fluids collected was 507 ml (500–800), and all fluids were clear. The pads were cut out with the attached parts of the intestine. The anastomotic area of the small intestine and the colon showed no sign of damage or leakage, and no fistula formation. The surface of the translucent serosa layer with the negative imprints of the film was seen with the aspect of a healing wound with macroscopic well-perfused tissue (Figure 4). In the same way, the bed of the gallbladder and untreated parts of the liver around were cut out, and the contact parts and free parts around of the pancreas were excised. All liver and pancreas surface areas showed no sign of pathology, the bed of the removed gallbladder was inconspicuous, and especially no sign of gall fistula formation was seen. All surfaces on which the film was applied showed the same pattern of protrusion as the pores of the film. These were typically missing in places that were not in contact with the film.

### Primary Endpoints (Histology)

The macroscopic described protrusion as found in all areas covered by the porous film was found in the same way in the histological cross sections and was made exclusively by serosa. Sparse granulocytic infiltration was seen in some areas. No signs of bleeding, no signs of thrombosis, or no other pathology was found in these areas. As a control, the uncovered surfaces showed no extensions and no other pathological changes.

**Figure 5:**
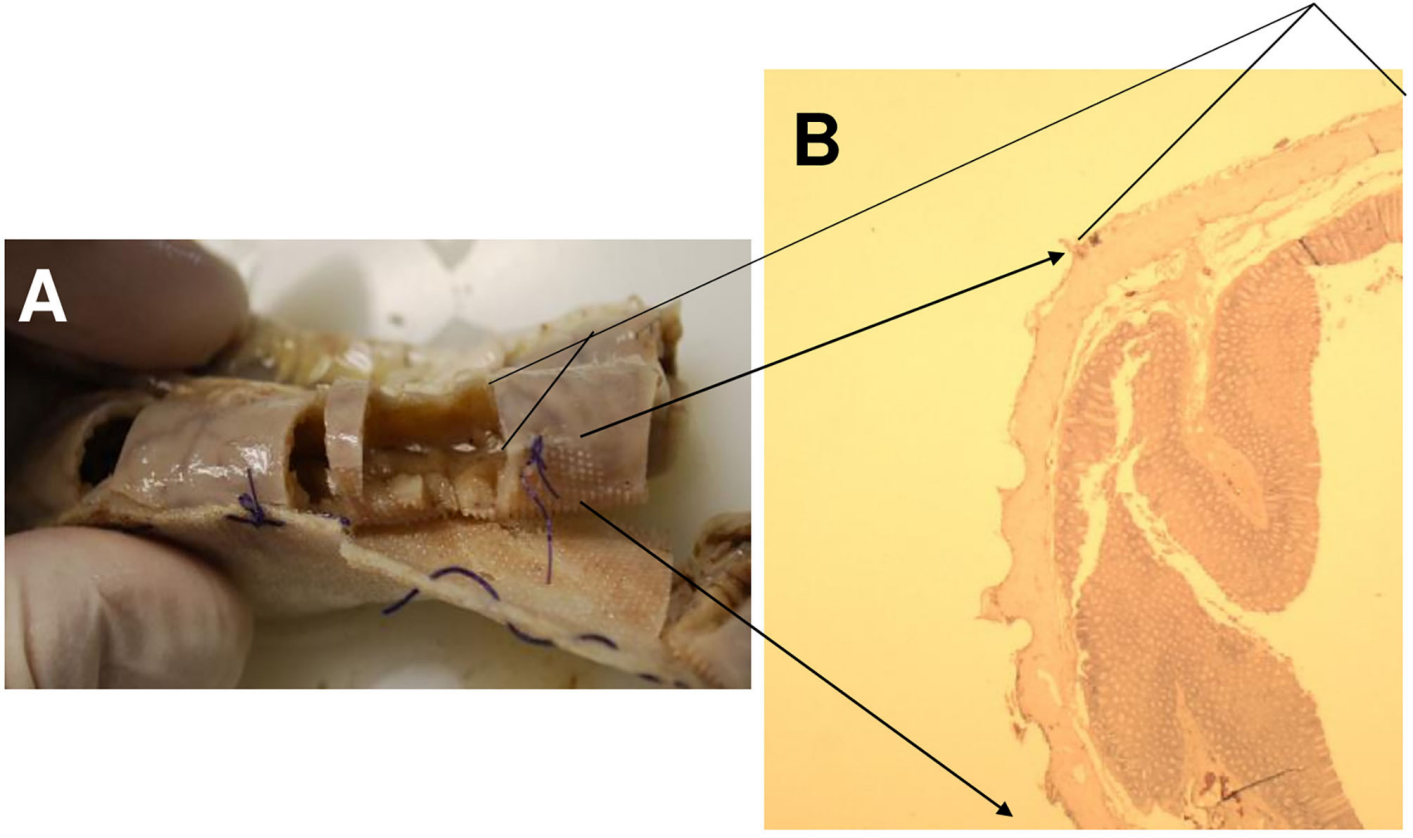
Animal 1, −80 mmHg. **(A)** Small intestine resection. Section after removal of the patch with a paving stone-shaped pattern (delimited by arrows). Non-patch area with smooth surface marked by bars. **(B)** Small intestine wall from the “patch” section near the anastomosis. Superficially with extensions (arrow) consisting of serosa, subserosa, and L. muscularis propria. This is with a paving stone-shaped surface (arrow). Underlying layers without pathological changes. Section marked with a bar from the non-patch area with a smooth surface (bar, corresponding to the bar in the macro illustration opposite).

**Figure 6:**
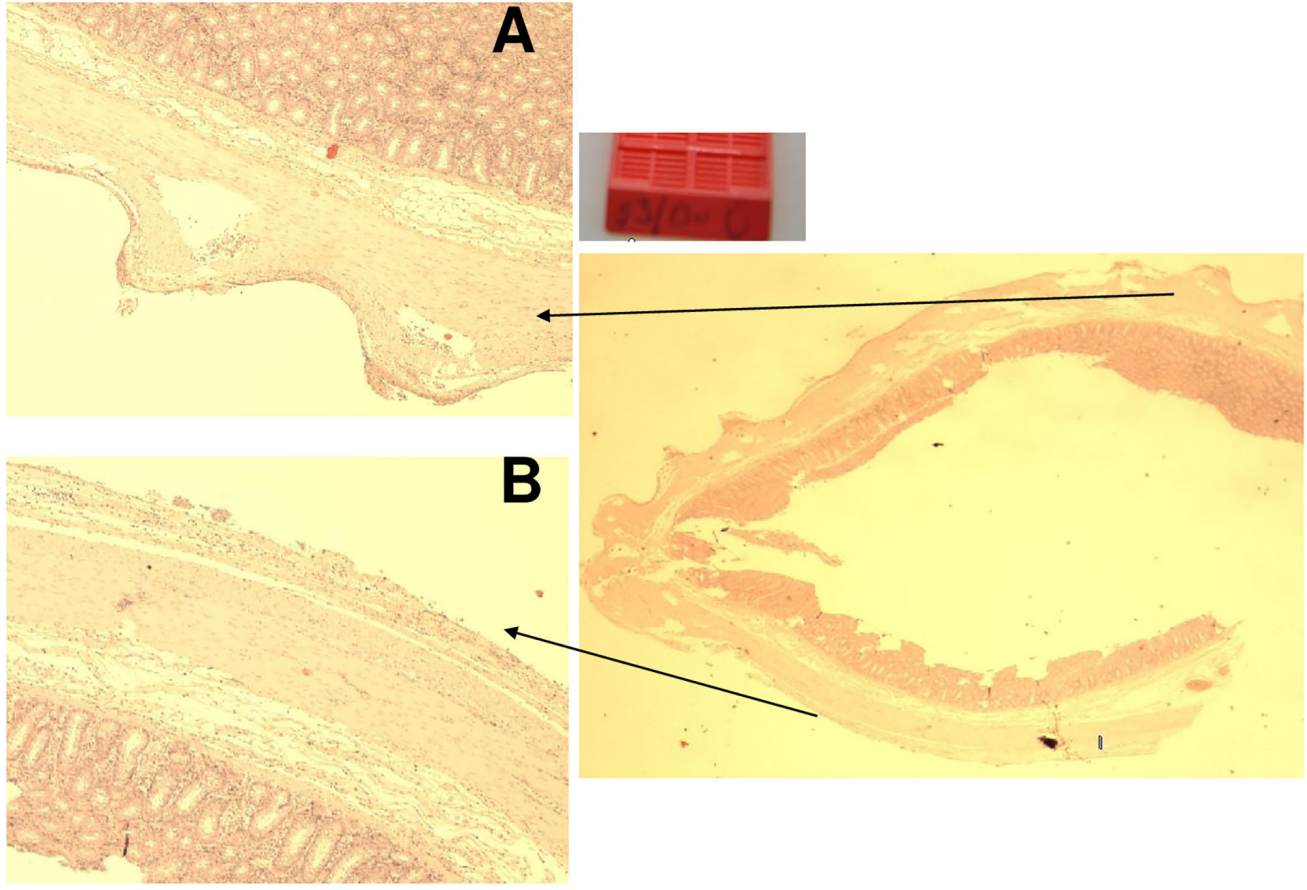
Animal 3, −80 mmHg. **(A)** Histology small intestine from the patch area. Extensions with serosa, subserosa, and lamina muscularis propria. Extended vessels in the lamina muscularis propria with intact structure (no rupture). **(B)** Histology from the non-patch area. Smooth surface.

**Figure 7:**
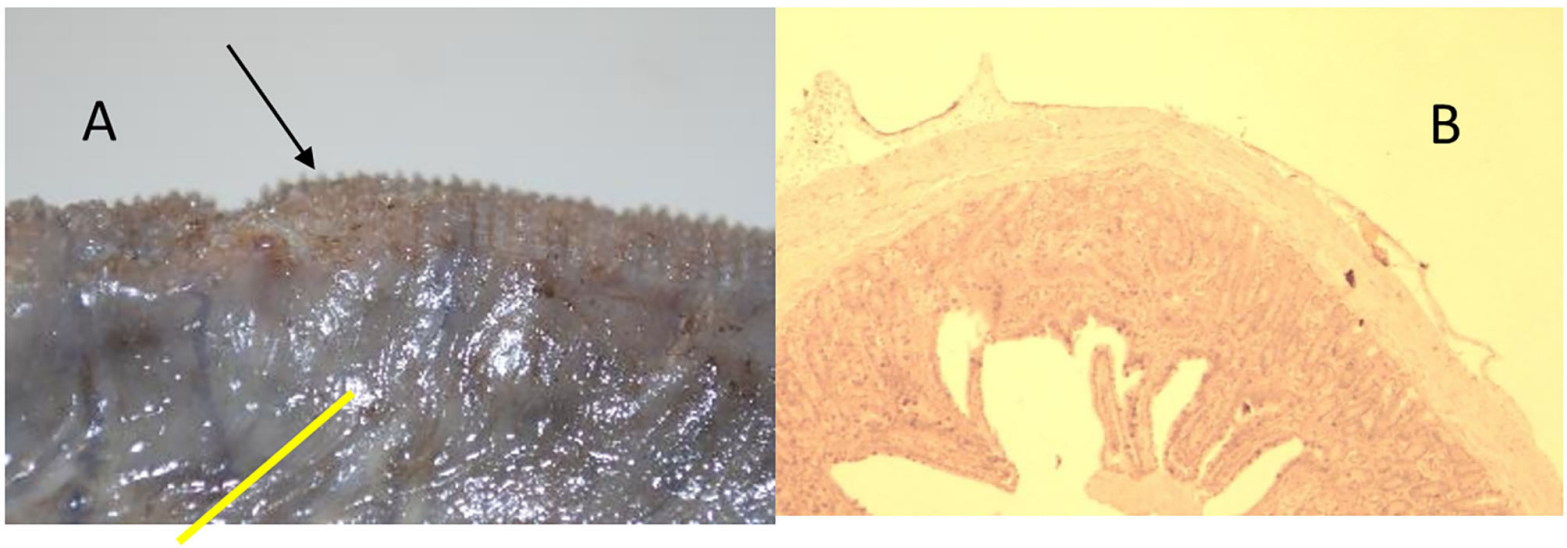
Animal 1, −80 mmHg. **(A)** Colon resection. Section after removing the patch with a paving stone-shaped pattern (arrows). Non-patch area with a smooth surface (yellow bar). **(B)** Colon wall from the “Patch” section. Superficially with extension consisting of serosa and subserosa. Underlying wall layers without pathological changes.

**Figure 8:**
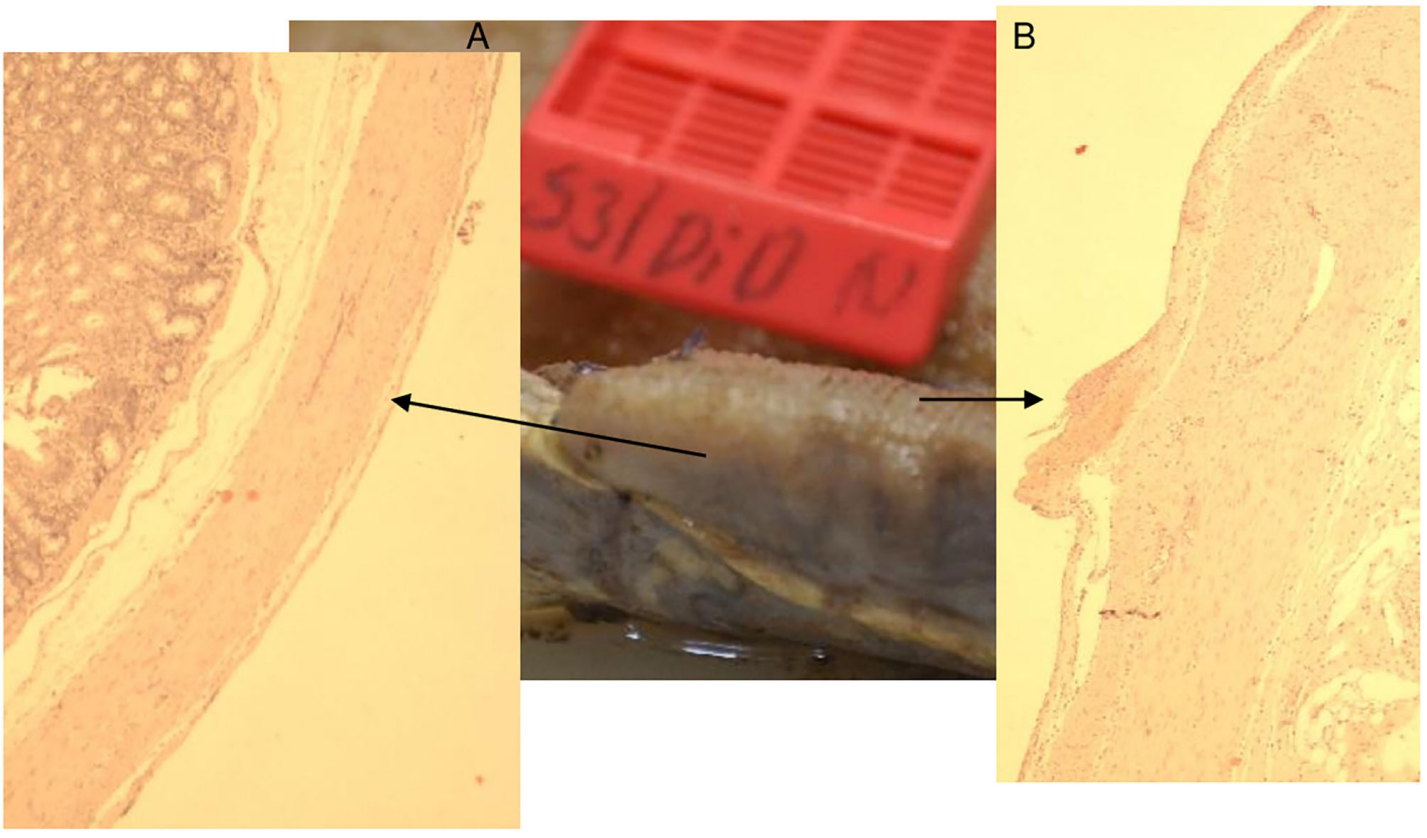
Animal 3, −80 mmHg Colon histology. **(A)** Without patch, smooth surface. **(B)** Spikes with serosa, subserosa, and hardly any lamina muscularis propria.

**Figure 9:**
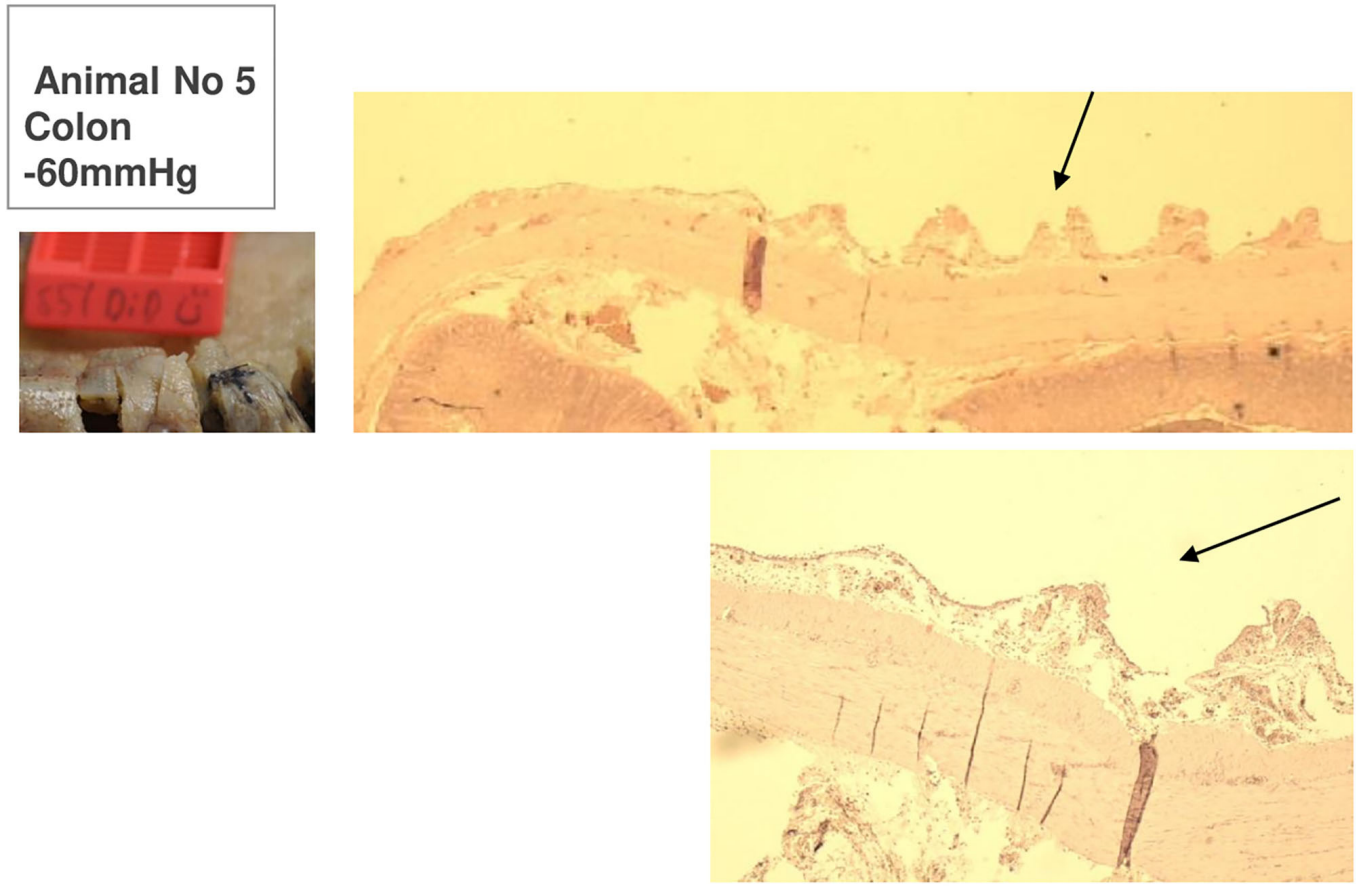
Animal 5, −60 mmHg. Colon histology. Transition patch and non-patch area. In the patch area (arrows) extensions of the serosa and subserosa. Lamina muscularis propria would not be pulled along.

**Figure 10:**
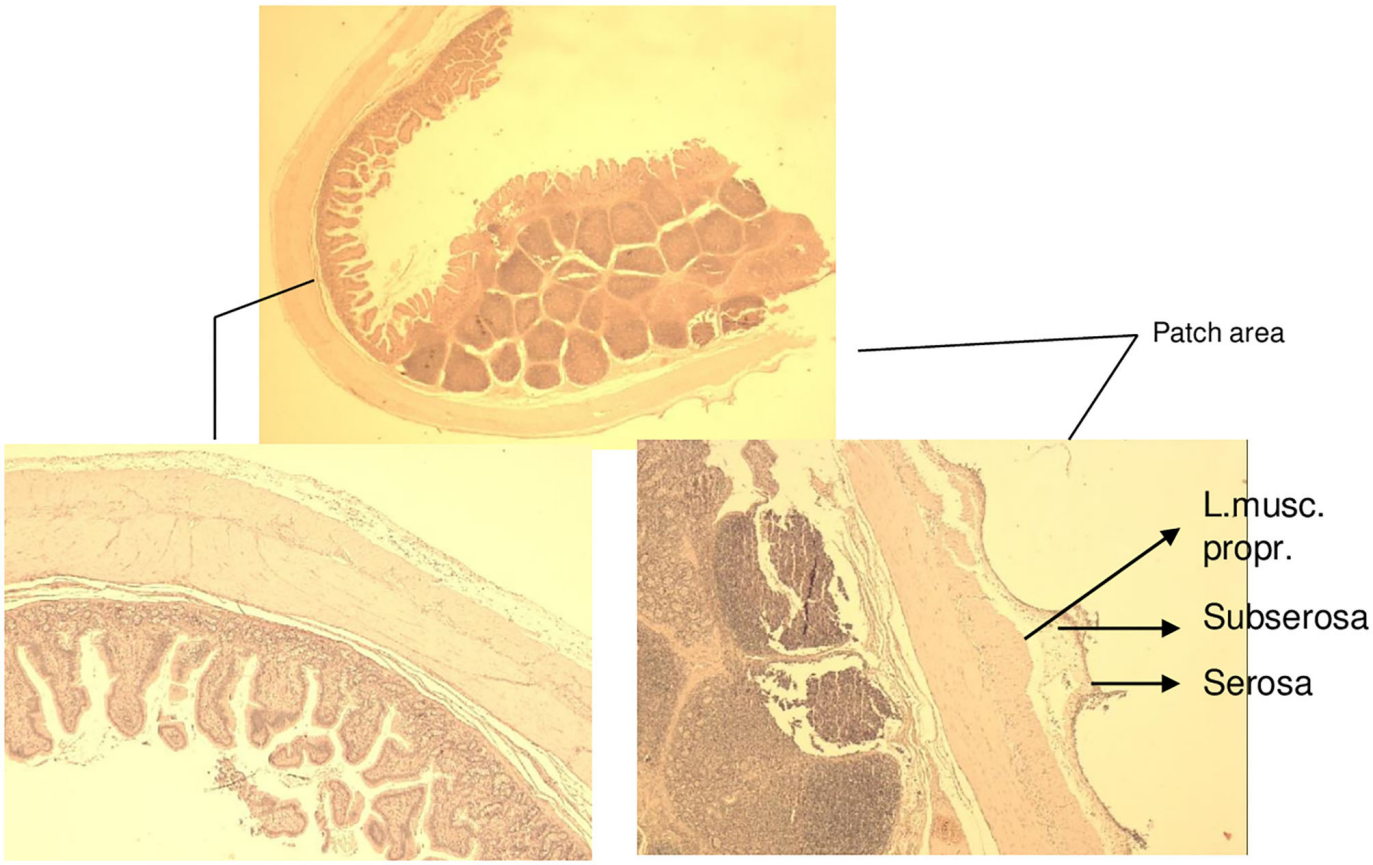
Animal 7, −60 mmHg. Histology small intestine. Patch to non-patch transition area. In the patch area, lobe-shaped extensions are mainly the serosa and subserosa affect. The lamina muscularis propria is only minimally involved. Smooth non-patch area surface.

**Figure 11:**
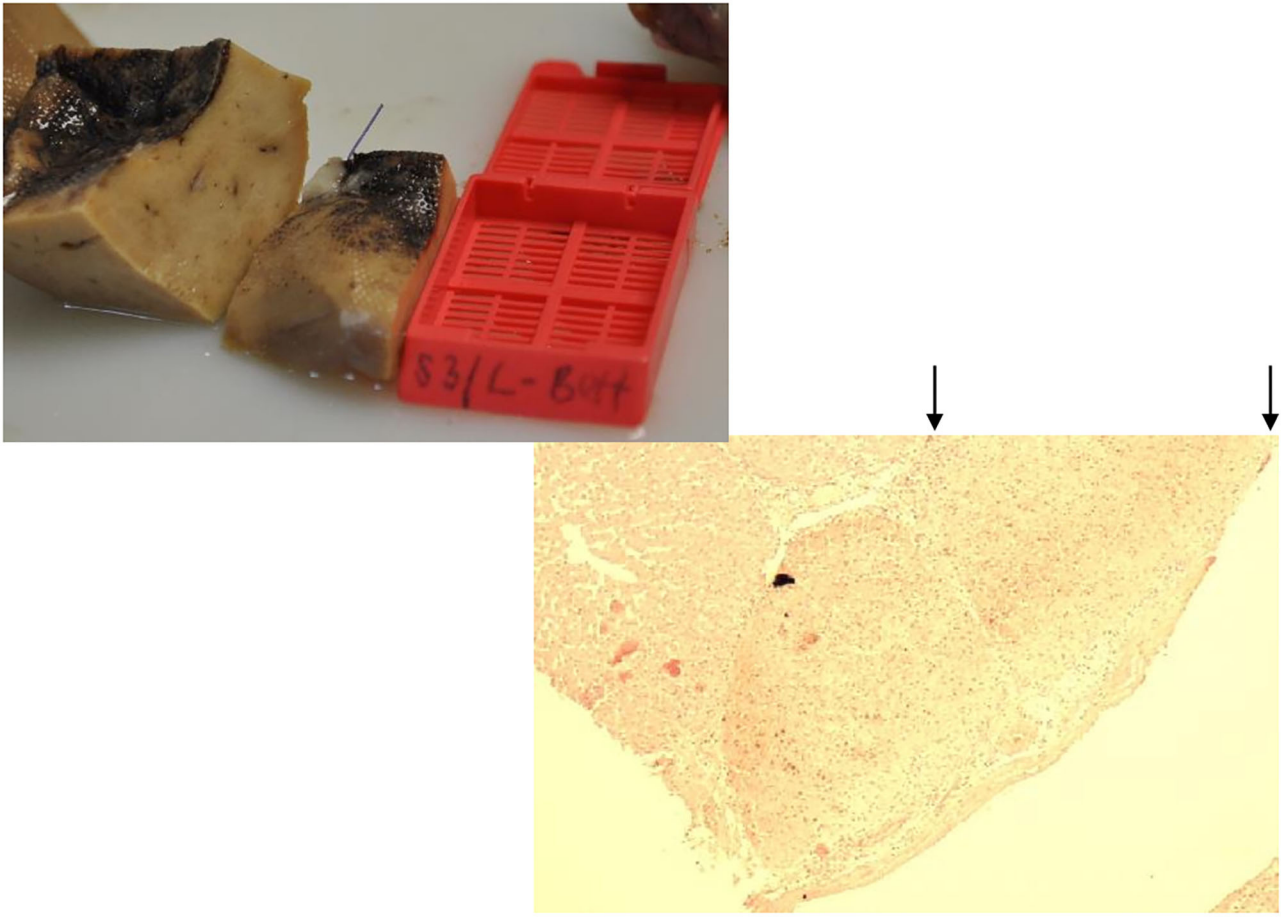
Animal 3, −80 mmHg. Histology from the liver bed: capsular and subcapsular inflammatory infiltrate with necrosis (marked by arrows). Intact liver parenchyma (bars).

**Figure 12:**
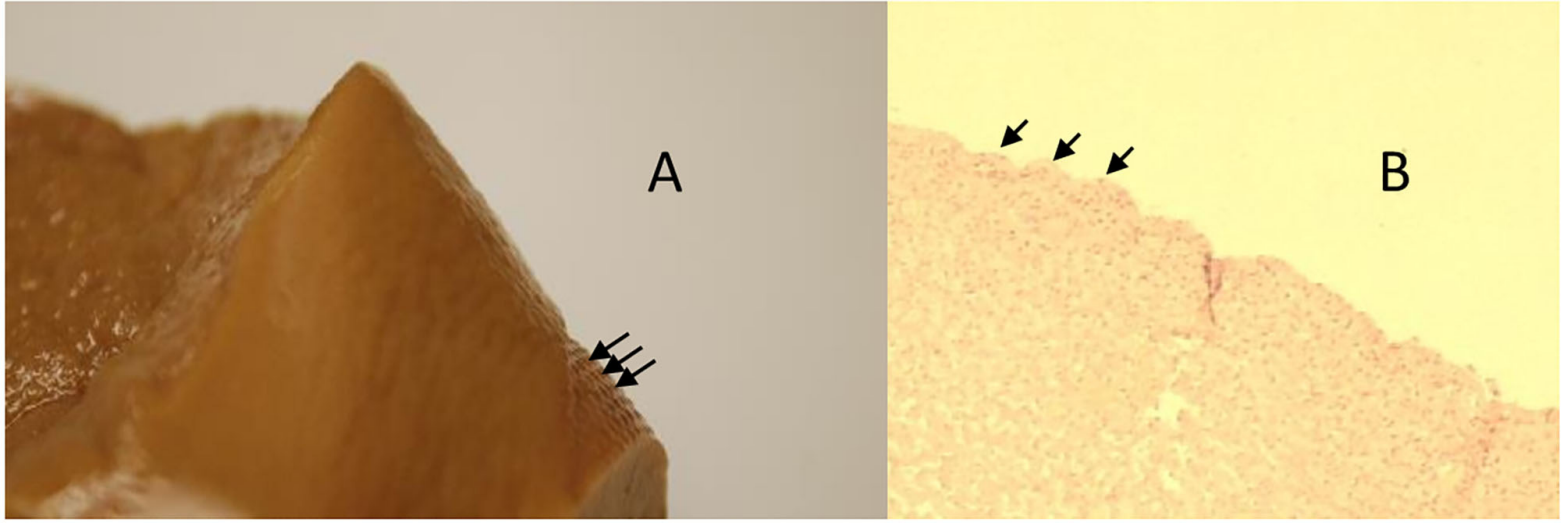
Animal 1, −80 mmHg. **(A)** Partial liver resection. Arrows mark areas with a paving stone surface, corresponding to the patched area. **(B)** Liver cut from the “patch” section. Superficially with a lobular shape (arrow) consisting of capsule and underlying, not pathologically altered liver cells.

**Figure 13:**
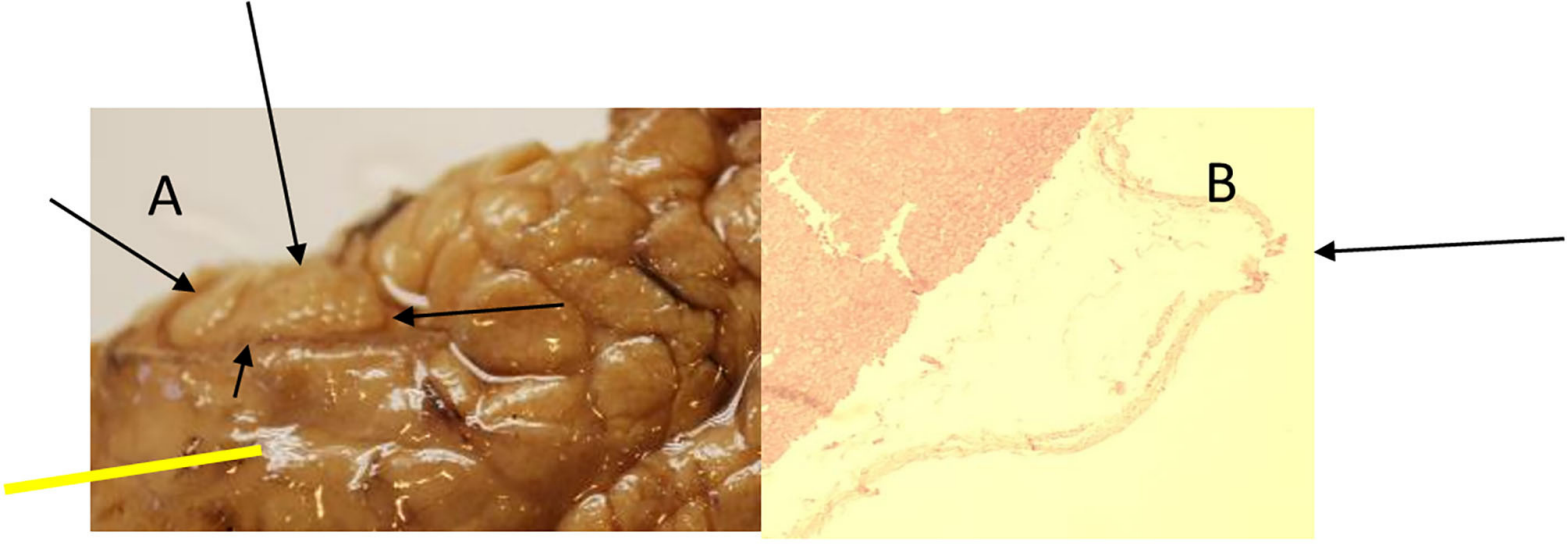
Animal 1, −80 mmHg. **(A)** Partial pancreatic resection. Arrows rewrite the section after patch removal with paving stone pattern. Non-patch area marked by bars and with a smooth surface. **(B)** Pancreas from “Patch” section. Superficial tip-shaped extension of the serosa coating (arrow). All underlying wall layers are regular without pathological changes. No inflammatory infiltrates.

The histological findings of the intestine, liver, and pancreas are described in Table 1. The investigated areas of anastomosis with pad and parts of them on the untreated surface did not give a different histological appearance (Figure 5). The difference between −80 and −60 mmHg was found on the examined small and large intestine wall. Whereas, extensions of the serosa and subserosa was found on all areas in contact with the film, extensions of the lamina muscularis propria was seen in nearly all histological specimens of intestine on animals treated with −80 mmHg. Especially on animal 3, extensions of blood vessels were also seen in the lamina muscularis propria plane of small intestine but were not accompanied by pathology such as thrombosis and rupture (Figure 6). In particular, the large intestine had less effect on the lamina muscularis propria than the small intestine with the −80-mmHg treatment, and no effect on the deeper wall layer similarly with small intestine with the −60-mm treatment (Figures 7–9). On animals 4–7, treated with −60 mmHg, the extensions of the lamina muscularis propria were found as spares, superficial, minimal, or none, on the histological sections of the intestine (Table 1, Figure 10). On all figures, parts without extensions represent areas without foil covering and are seen as a control. These areas showed an intact serosa form and no histological changes to the wall layers of the small intestine and large intestine. Histological findings on liver sections were typical for findings after removal of the gallbladder: edema, necrosis, and granulocytic infiltration (Figure 11). Around the resection bed and on the surface in contact with suction pads, the findings were such as sparse lymphocytic infiltration and extension of the serosa capsule, but unchanged liver tissue (Figure 12). The portions of the liver without a film covering showed no pathological changes whatsoever on the liver surface or on the underlying parenchyma. Similar findings were found on the pancreas surface: extensions of the serosa capsule on the film contact side and unchanged pancreas tissue on all sections (Figure 13). In particular, no sign of the onset of pancreatitis was found. The parts not covered with the film showed no capsular expansion and no pathological changes in the pancreatic parenchyma.

**Table 1:** Histological findings for small bowel, colon, liver, and pancreas of animals 1–7, indicating corresponding negative pressure.

**Animal number**	**Negative pressure**	**Small bowel**	**Colon**	**Liver**	**Pancreas**
1	−80 mmHg	Extension of: serosa, subserosa, Lamina muscularis propria	Extension of: Serosa, subserosa	Extension of: Serosa, Granulocytic infiltration	Extension of: Serosa
2	−80 mmHg	Extension of: Serosa, subserosa Partially: Lam.muscul.propr. Sparse lymphocutic infiltr	Extension of: Serosa, Subserosa, Lam. muscul. propria	Gallbladder bed: necrosis, Subcapsulary edema	Extension of: Serosa
3	−80 mmHg	Extension of: Serosa, Subserosa, Lam.muscularis propria Blood vessels (no structural damage)	Extension of: Serosa, subserosa, Lam. muscul. propria	Gallbladder bed: necrosis, Granulocytic infiltration	Extension of: Serosa
4	−60 mmHg	Extension of: Serosa Partially subserosa Sparse superficial parts of the Lam.muscularis propria	Extension of: Serosa	Sparse granulocytic infiltration	Sparse granulocytic infiltration
5	−60 mmHg	Extension of: Serosa, subserosa Very sparse parts of the Lam.muscularis propria	Extension of: Serosa, subserosa	Sparse granulocytic infiltration	Sparse granulocytic infiltration
6	−60 mmHg	Extension of: Serosa, subserosa Partially Lam.muscularis propria	Extension of: Serosa, subserosa Partially Lam.muscularis propria	Subcapsulary edema of the gallbladder bed	Serosa extensions
7	−60 mmHg	Extension of: Serosa, subserosa Minimal parts of Lam.muscularis propria	Extension of: Serosa, subserosa Isolated superficial parts of Lam.muscularis propria	Gallbladder bed: Subcapsulary edema	Serosa extensions

## Discussion

For this experiment, a compromise had to be found between ethical feasibility, costs, and meaningfulness. It is believed that this was found in a full day's experiment with small domestic pigs under general anesthesia over the entire term. Open-abdomen NP therapy has a minimal duration of 24–48 h, and treatments can last up to several weeks. The results of this 8-h study must be seen as approximations of a much more sensitive but very comparable system (11) in relation to human tissue. The small intestine, for example, has an empty diameter of ~1 cm and the wall diameter half of a human small intestine (12). In addition, the direct pad-to-intestine contact was reinforced by the direct cable routing to the suction pump, in contrast to the distribution effect when the film was placed on the intestine over a large area. Thus, in our opinion, the application of −60 and −80 mmHg also has a much stronger impact after a shorter period of time and allows conclusions to be drawn about the expected effects of longer applications on human tissue. Studying the development of a fistula formation of the intestine, first signs as microcirculatory disturbance and signs of necrosis are to be expected (8). As a discreet sign of an incipient microcirculation disorder, we already assessed the pulling out of a vessel with the lamina propria, only seen in test animal 3, but also the clear pulling out of only the lamina propria muscularis, even if this remained without any sign of a functional disorder such as thrombosis or necrobiosis. As these significant changes only occur at −80 mmHg, this was set as the maximum end point for the strength of the suction for further therapeutic use in humans. At −60 mmHg, the described effects were limited mainly on the serosa and subserosa in the form of extracts in the pore pattern of the film, so this was set as the standard pressure for the therapeutic use of the film. We see these results in certain contradiction to the observations of the Lindstedt group: significant reduction in microcirculation of the small bowel loops and omentum with application of NP from −50 to −170 mmHg. A reduction of this effect could be achieved by placing a protection plate. A dependency of the interference effect on the microcirculation was found depending on the distance of the pressure buildup system (8, 13). We also see a discrepancy of these microcirculatory findings with the results of Bjarnason et al. (9); only minimal residual NP was observed below the AB-Thera^R^ system of applied NP from −50 to −150 mmHg. The main differences to our experiment were the film used (Suprasorb CNP^R^ vs. AB-Thera/V.A.C^R^) and the observation window (histology vs. blood flow determination vs. pressure measurement). The authors of the study (8), Hlebowicz et al., estimated the occasional association of the observed decrease of blood flow with ischemia, promoting the development of intestinal fistulae. Two models are considered as hypotheses: the effect of NP *per se*, which decreases with the distance of the tissue from the application and is more pronounced in soft tissue than in the firm one. On the other hand, based on the effects of the NP application in proximity to the heart muscle, a model of herniation in the direction of the NP application is considered. In our model, the suction application was applied directly to the intestinal loop, and the suction pump was also connected to a direct line. This means on the one hand a very intensive contact directly with the intestinal surface, on the other hand a relatively stable system that hardly leaves any freedom of movement, even if the highest suction power was less than half that of the experiment by Hlebowicz et al. (8). From this point of view, we would tend to the herniation model and assume that the measured perfusion reductions were caused by an incarceration effect or by kinking of vessels. Because, if a perfusion disorder were caused by contact alone, damage would have to be visible histologically in our model after 8 h and also with −60 mmHg. The effect of a protective disc presented in the publication by Lindstedt et al. (14) could confirm this assumption, since it may act as a support for the bowel loop. In any case, it would be interesting to examine our two models together.

The application of the suction pads to the gallbladder bed of the liver after cholecystectomy had no effects on the liver tissue apart from the known serosa extractions, i.e., it showed no differences under the two selected suction settings in comparison to the free liver surface. The same result was also seen in the pancreatic tissue. To our astonishment, this tissue, which is otherwise sensitive to manipulation, showed no effects on the parenchyma itself, with the exception of the serosa extensions mentioned several times. These observations in comparison with the abovementioned perfusion model would in turn underline the hernia model, since both organs and especially the pancreatic tissue would have to show effects of direct negative pressure on the perfusion in the histomorphology. The truth could be in between. With our model, we come to a significant reduction in the suggested suction strength of −60 mmHg to the widespread use of −120 mmHg, but with a completely different structure of the film used. Possibly a direct influence on perfusion, especially in the intestinal wall, begins even with stronger suction values of −80 mmHg and more. Lindstedt et al. (14) describe only a slight protective effect of the protective disc at −120 mmHg compared to a clear effect at −50 mmHg: large protective effect through the stabilization of the disc with little pressure, little effect against the strong NP *per se*.

As conclusions from our results, the application of −60 mmHg is given a guide value for the use of the Suprasorb-CNP system in the abdomen. In any case, the value of −80 mmHg should never be exceeded, if intestinal tissue comes into contact with the foil tested in this study.

The following clinical studies will have to show whether this system can represent an extension of the spectrum of NP therapy. Many areas of septic abdomen treatment, deep abscess treatment in the abdomen, necrotizing pancreatitis, anastomotic treatment in infectious environment, and also fistula treatment leave a lot to be desired. We hope to be able to provide an impetus through this study.

## Data Availability Statement

The raw data supporting the conclusions of this article will be made available by the authors, without undue reservation.

## Ethics Statement

The animal study was reviewed and approved by Austrian Ministry, according to animal testing law (BGBI.Nr.501/1988 i.d.g.F.).

## Author Contributions

All authors listed have made a substantial, direct and intellectual contribution to the work, and approved it for publication.

## Conflict of Interest

The authors declare that the research was conducted in the absence of any commercial or financial relationships that could be construed as a potential conflict of interest.
